# Supplementary material for: Detection of Exotic Mosquito Species (Diptera: Culicidae) at International Airports in Europe
Source: Int J Environ Res Public Health. 2020 May 15;17(10):3450. doi: 10.3390/ijerph17103450 (PMC7277938; doi:10.3390/ijerph17103450)
Supplement: Supplementary file 1 [file ijerph-17-03450-s001.pdf]

Supplementary material

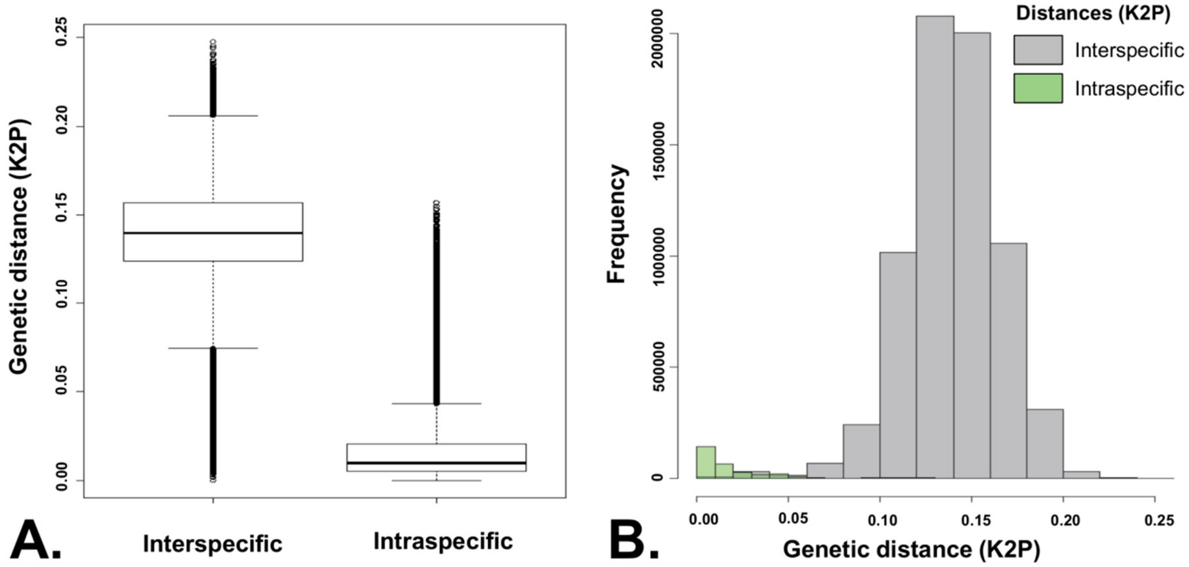

Figure S1. Evaluation of barcode gap in the dataset (species listed in Table S3, 2,721 COI sequences included). A. Boxplot of the inter- and intraspecific genetic distances, indicating the existence of a barcode gap, with interspecific distance being larger than intraspecific distance. The median is indicated by the horizontal line and the range as the vertical dashed lines (upper and lower 1.5 IQR quartiles). Outliers are displayed as open circles (due to overlap, they look like bold vertical lines). Box represents the upper and lower quartiles; B. Frequency of pairwise comparisons against base substitution per site (K2P).

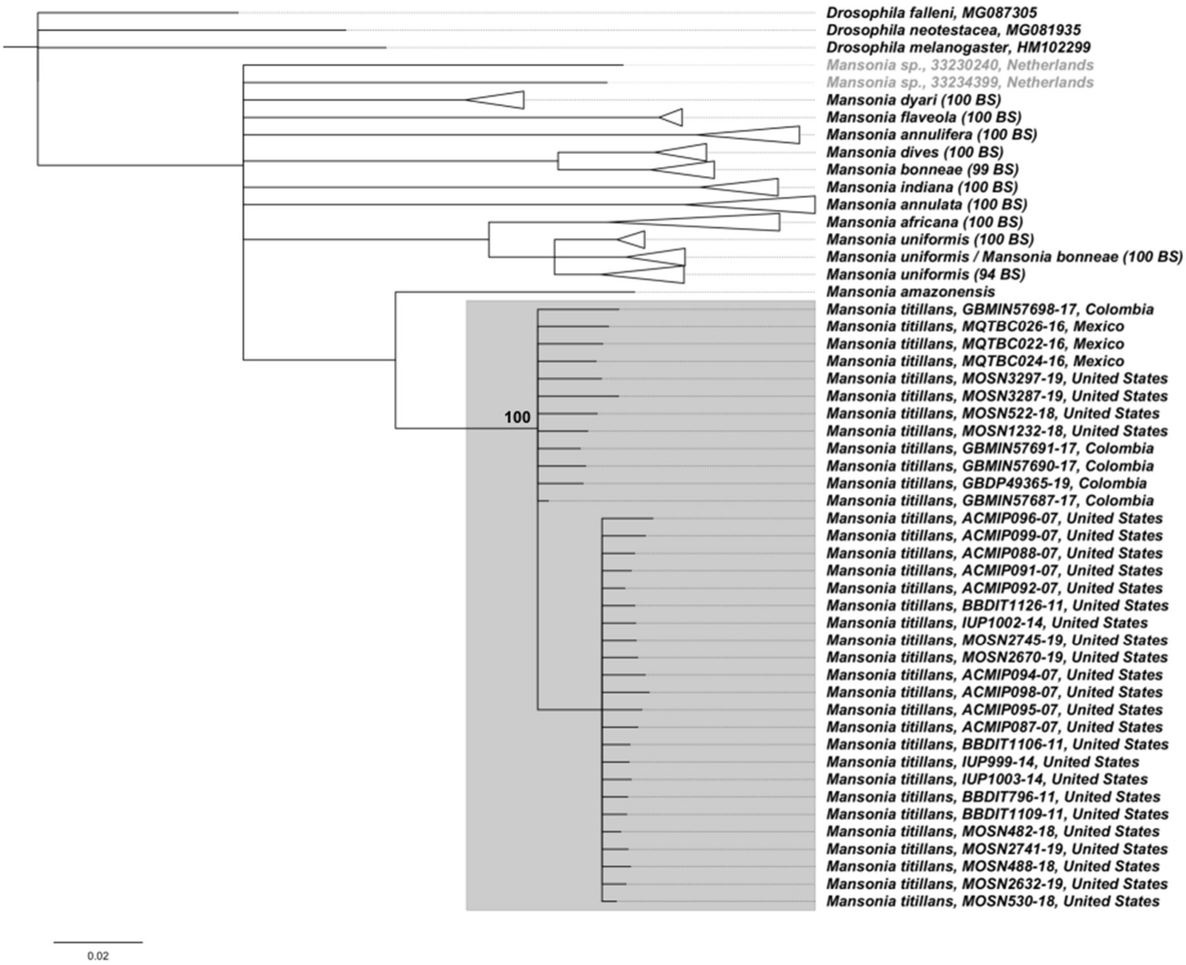

Figure S2. Neighbour-Joining tree including 11 *Mansonia* species based on COI (K2P; 658 bp fragment; 227 barcodes). The bootstrap values (BS; 500 replicates) are shown in the tip labels for the collapsed sequence clusters, while they are displayed at the branch points for the non-collapsed species (sequences of query specimens highlighted in grey). Clusters were collapsed to make the overall tree visually clearer. Minimum bootstrap displayed is 75, other branches are collapsed.

Table S1. Table reporting the primers involved for the amplification of the three DNA regions. References in the manuscript.

Primer	Sequence	Reference	DNA region
LCO1490	5'-GGTCAACAAATCATAAAGATATTGG-3'	Folmer *et al.* 1994	Mitochondrial COI
HCO2198	5'-TAAACTTCAGGGTGACCAAAAAATCA-3'	Folmer *et al.* 1994	Mitochondrial COI
5.8S	5′-TGTGAACTGCAGGACACATG-3′	Collins and Paskewitz 1996	Nuclear ITS2
28S	5′-ATGCTTAAATTTAGGGGGTA-3′	Collins and Paskewitz 1996	Nuclear ITS2
N4J-8502D	5′-CGTAGGAGGAGCAGCTATATT-3′	Fonseca *et al.* 2001	Mitochondrial nad4
N4N-8944D	5′-AAGGCTCATGTTGAAGCTCC-3′	Fonseca *et al.* 2001	Mitochondrial nad4

Table S2. PCR cycling conditions used for the amplification of the three DNA regions.

	Initial denaturation	Denaturation	Annealing	Elongation	Final elongation	Cycles
COI	94 °C - 3 min	94 °C - 30 s	45 °C - 30 s	72 °C - 60 s		5
		94 °C - 30 s	51 °C - 60 s	72 °C - 60 s	72 °C - 10 min	35
ITS2	94 °C - 3 min	94 °C - 30 s	46 °C - 30 s	72 °C - 45 s	72 °C - 7 min	35
nad4	94 °C - 3 min	94 °C - 30 s	55 °C - 40 s	72 °C - 50 s	72 °C - 10 min	35

Table S3. List of mosquito species occurring in Belgium and the Netherlands, including the overall maximum observed intraspecific Kimura two-parameter (K2P) distances among COI sequences. The K2P values in brackets were calculated based on a database that included the four query COI sequences. The checklist of species was compiled from a literature review, using the publications of [4], [23]–[32], and includes the four new species identified within this study (highlighted in bold). (*) indicates the sequences were extracted from GenBank. If not specified, sequences were extracted from BOLD (public). n.a.: not applicable.

Subfamily	Tribe	Genus	Subgenus	Species	Belgium	Netherlands	Number of COI sequences included in the analyses	Average inter-specific K2P distance (%)	Max observed K2P distance between conspecific sequences (%)
Anophelinae		*Anopheles* Meigen, 1818	*Anopheles* Meigen, 1818	*algeriensis* Theobald, 1903		√	8	12.19	6.33
				atroparvus Van Thiel, 1927	√	√	18	2.49	1.51
				claviger (Meigen, 1804)	√	√	14	11.20	3.83
				***crucians* Wiedemann, 1828**		√	**21 (22)**	**8.15 (8.14)**	**5.30 (5.50)**
				maculipennis Meigen, 1818	√	√	14	2.28	2.71
				melanoon Hackett, 1934		√	1*	1.86	n.a.
				messeae Falleroni, 1926	√	√	75	2.73	2.81
				***pharoensis* Theobald, 1901**	√		**4 (5)**	**12.95 (12.98)**	**1.32 (1.75)**
				plumbeus Stephens, 1828	√	√	13	13.76	0.47
				***subpictus* Grassi, 1899**		√	**65 (66)**	**10.86 (10.86)**	**14.72 (14.72)**
Culicinae	Aedini	*Aedes* Meigen, 1818	*Aedes* Meigen, 1818	*aegypti* (Linnaeus, 1762)		√	158	9.21	3.09
				cinereus Meigen, 1818	√	√	307	11.84	5.56
			Aedimorphus Theobald, 1903	*vexans* (Meigen, 1830)	√	√	437	0.97	4.62
			Dahliana	*geniculatus*	√	√	15	9.88	1.71

			Reinert, Harbach & Kitching, 2006	(Olivier, 1791)					
			Georgecraigius Reinert, Harbach & Kitching, 2006	*atropalpus* (Coquillett, 1902)		√	5	9.76	1.14
			Hulecoeteomyia Theobald, 1904	*japonicus* (Theobald, 1901)	√	√	52	9.44	9.65
				koreicus (Edwards, 1917)	√		6	7.05	0.74
			Ochlerotatus Lynch Arribálzaga, 1891	*annulipes* (Meigen, 1830)	√	√	12	1.01	2.01
				cantans (Meigen, 1818)	√	√	10	0.33	1.30
				caspius (Pallas, 1771)	√	√	27	1.98	2.83
				communis (De Geer, 1776)	√	√	207	2.85	5.68
				detritus Haliday, 1833	√	√	4	7.74	1.11
				dorsalis (Meigen, 1830)	√	√	95	1.31	1.26
				excrucians (Walker, 1856)		√	80	0.85	2.45
				flavescens (Müller, 1764)	√	√	17	2.61	5.97
				leucomelas (Meigen, 1804)		√	2	8.72	0.40
				nigrinus (Eckstein, 1918)		√	2	0.30	0.77
				punctor (Kirby, 1837)	√	√	31	4.61	5.85
				riparius Dyar & Knab, 1907		√	7	4.53	2.20
				sticticus (Meigen, 1838)	√	√	250	5.86	10.76
				rusticus (Rossi, 1790)	√	√	4	6.97	0.27
			Stegomyia Theobald, 1901	*albopictus* (Skuse, 1895)	√	√	215	11.09	12.38
	Culicini	*Culex* Linnaeus, 1758	*Barraudius* Edwards, 1921	*modestus* Ficalbi, 1890		√	33	5.45	4.74
			Culex Linnaeus, 1758	*pipiens* Linnaeus, 1758	√	√	103	2.19	7.63
				torrentium Martini, 1925	√	√	99	1.82	0.90
			Neoculex Dyar, 1905	*territans* Walker, 1856	√	√	181	9.43	6.63
			Maillotia Theobald, 1907	*hortensis* Ficalbi, 1889	√		9	8.87	0.85
	Culisetini	*Culiseta* Felt, 1904	*Culicella* Felt, 1904	*fumipennis* (Stephens, 1825)	√	√	5	4.15	9.04
				morsitans (Theobald, 1901)	√	√	76	6.83	9.99
				ochroptera Peus, 1935		√	2	10.50	2.27
			Culiseta Felt, 1904	*alaskaensis* (Ludlow, 1906)		√	9	4.09	0.63
				annulata (Schrank, 1776)	√	√	10	2.13	0.27
				subochrea (Edwards, 1921)	√	√	1	2.01	n.a.
	Mansoniini	*Coquillettidia*	*Coquillettidia*	*richiardii*	√	√	10	13.31	0.55

		Dyar, 1905	Dyar, 1905	(Ficalbi, 1889)					
	Orthopodomyiini	*Orthopodomyia* Theobald, 1904	*Orthopodomyia* Theobald, 1904	*pulcripalpis* (Rondani, 1872)	√		1*	12.05	n.a.

Table S4. Results of the species identifications obtained using the BOLD and BLAST (GenBank) search engines. Nad4 blasting results were inconclusive because there are presently no nad4 sequences of the identified species (based on COI) in the online repository GenBank. There are at present no published COI sequences of *Anopheles crucians* in GenBank. n.a.: no sequencing product.

Sample ID	BOLD species match and similarity %	BLAST (GenBank) species match and similarity %		
	COI		ITS2	nad4
05894947	*Anopheles crucians* 99.60%	*Anopheles sp.* 92.70%	*Anopheles crucians* 99.68%	Inconclusive < 90%
05899545	*Anopheles subpictus* 100%	*Anopheles subpictus* 100%	*Anopheles subpictus* 99.22%	Inconclusive < 90%
33230240	Inconclusive < 90%	Inconclusive < 90%	Inconclusive < 90%	Inconclusive < 90%
33234399	Inconclusive < 90%	Inconclusive < 90%	Inconclusive < 90%	n.a.
M17M0017	*Anopheles pharoensis* 99.08%	*Anopheles pharoensis* 99.09%	*Anopheles pharoensis* 99.63%	Inconclusive < 90%
